# Development of SNAP-Tag Fluorogenic Probes for Wash-Free Fluorescence Imaging

**DOI:** 10.1002/cbic.201100173

**Published:** 2011-07-26

**Authors:** Xiaoli Sun, Aihua Zhang, Brenda Baker, Luo Sun, Angela Howard, John Buswell, Damien Maurel, Anastasiya Masharina, Kai Johnsson, Christopher J Noren, Ming-Qun Xu, Ivan R Corrêa

**Affiliations:** [a] New England Biolabs, Inc240 County Road, Ipswich, MA 01938 (USA); [b] Institute of Chemical Sciences and Engineering, Ecole Polytechnique Federal de Lausanne (EPFL)1015 Lausanne (Switzerland)

**Keywords:** cell imaging, covalent labeling, fluorescent probes, fluorogenic substrates, protein modifications

## Abstract

The ability to specifically attach chemical probes to individual proteins represents a powerful approach to the study and manipulation of protein function in living cells. It provides a simple, robust and versatile approach to the imaging of fusion proteins in a wide range of experimental settings. However, a potential drawback of detection using chemical probes is the fluorescence background from unreacted or nonspecifically bound probes. In this report we present the design and application of novel fluorogenic probes for labeling SNAP-tag fusion proteins in living cells. SNAP-tag is an engineered variant of the human repair protein *O*^6^-alkylguanine-DNA alkyltransferase (hAGT) that covalently reacts with benzylguanine derivatives. Reporter groups attached to the benzyl moiety become covalently attached to the SNAP tag while the guanine acts as a leaving group. Incorporation of a quencher on the guanine group ensures that the benzylguanine probe becomes highly fluorescent only upon labeling of the SNAP-tag protein. We describe the use of intramolecularly quenched probes for wash-free labeling of cell surface-localized epidermal growth factor receptor (EGFR) fused to SNAP-tag and for direct quantification of SNAP-tagged β-tubulin in cell lysates. In addition, we have characterized a fast-labeling variant of SNAP-tag, termed SNAP_f_, which displays up to a tenfold increase in its reactivity towards benzylguanine substrates. The presented data demonstrate that the combination of SNAP_f_ and the fluorogenic substrates greatly reduces the background fluorescence for labeling and imaging applications. This approach enables highly sensitive spatiotemporal investigation of protein dynamics in living cells.

## Introduction

The ability to study the dynamic functions of proteins in living cells has been greatly aided by the development and application of tagging tools.[1, 2] An emerging technique for live-cell imaging and proteomics applications is the site-specific labeling of cellular proteins with chemical probes.[3–6] In this approach, small organic molecules are coupled to the protein being studied via a fusion tag, either by self-labeling or enzymatic ligation. Several different peptide and protein fusion tags have been developed to study proteins in living systems, including the tetracysteine tag,[7] HaloTag,[8] TMP-tag,[9] β-lactamase-tag,[10] ACP-tag,[11] BirA acceptor peptide,[12] and LplA acceptor peptide.[13] Among the most prominent fusion tags is the SNAP-tag, an engineered variant of the human repair protein *O*^6^-alkylguanine-DNA alkyltransferase (hAGT) that covalently reacts with *O*^6^-benzylguanine (BG) derivatives bearing a chemical or optical probe.[14, 15] During the reaction with a substrate, a stable thioether bond is formed between the reactive cysteine of the tag and the label. SNAP-tag reactions proceed with a well-defined mechanism, predictable stoichiometry and rapid kinetics, irrespective of the fusion protein attached to the tag. SNAP-tag labeling offers a variety of advantages over traditional tagging of proteins using autofluorescence. In addition to labeling by fluorescent probes, SNAP-tag fusion proteins can be modified with affinity ligands or other binding moieties,[16] used for selective crosslinking of interacting protein partners,[17, 18] immobilization on solid surfaces for purification, pull-downs, and protein microarray experiments,[19] and allows temporal control over labeling. All these features provide an additional level of sophistication and flexibility for assessing protein function and dynamics in cell biology. The utility of the SNAP-tag self-labeling technology has been demonstrated for the study of protein localization and trafficking in live mammalian cells.[20, 21]

Despite the ease of temporal control and broad range of commercially available fluorescent probes, widespread use of SNAP-tag for visualization and study of protein dynamics is limited by background fluorescence from unreacted or nonspecifically bound substrates. Klein et al. have recently reported that coating the glass chambers with glycine prior to seeding cells minimizes the nonspecific adsorption of fluorophore conjugates on glass surfaces for super-resolution imaging applications.[22] However, as for the vast majority of chemical labeling approaches, a thorough wash step is still required to reduce fluorescence signals due to the presence of unreacted probes. Besides being a tedious and time-consuming process, this requirement may potentially limit some applications, such as direct quantification of protein concentration in cell lysates or real-time monitoring of molecular events like receptor-ligand binding, endocytosis, trafficking, and expression of newly synthesized proteins. Thus a strong need remains for efficient molecular imaging methods that enable researchers to access real-time detection and high-contrast imaging.

**Figure 1:**
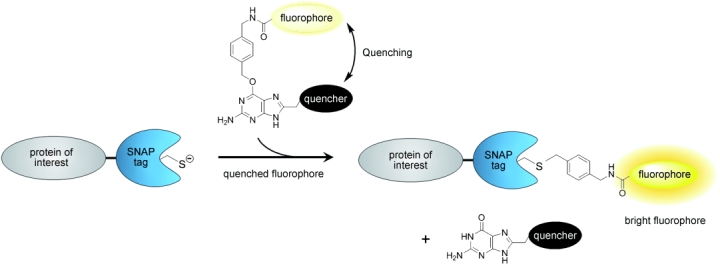
Labeling of SNAP-tag fusion proteins with fluorogenic probes.

Herein, we report the design and application of intramolecularly quenched (“dark”) fluorogenic benzylguanine probes that become highly fluorescent upon reaction with a SNAP-tag. The utility of this approach has been very recently demonstrated by Komatsu et al. using SNAP-tag technology and three activatable fluorescent probes to conduct real-time measurements of protein dynamics.[23] We further advance the value of SNAP-tag labeling technology by combining a faster labeling variant of SNAP-tag, termed SNAP_f_, with a broader range of fluorogenic benzylguanine probes for wash-free labeling of fusion proteins in living cells. Our strategy was to explore different combinations of fluorophore/quencher pairs in an attempt to obtain optimal intramolecularly quenched substrates with fluorescence emission across the visible spectrum, while retaining a rapid reaction rate between the probe and fusion protein tag (Figure 1).

Fluorogenic substrates generate an intense fluorescence signal only after reaction with their targets, therefore greatly reducing background fluorescence.[10, 24, 25] The high signal-to-noise ratio of the fluorogenic substrates enables wash-free assays, and as a consequence, facilitates high-throughput screening[26–28] and real-time analysis of dynamic cellular processes, such as protein expression, localization, trafficking and degradation.[29–31] In order to demonstrate the utility of SNAP_f_ and these self-quenching probes, we designed an assay to measure the concentration of proteins in cell extracts and demonstrated wash-free, high-contrast imaging of plasma membrane localization of the epidermal growth factor receptor (EGFR) in living HEK293 cells. We also describe a colocalization study of SNAP_f_-EGFR with epidermal growth factor ligand fused to a fast labeling version of the related CLIP-tag[32] protein (EGF-CLIP_f_).

## Results and Discussion

### Design and synthesis of fluorogenic substrates

SNAP-tag fluorogenic probes consist of benzylguanine substrates bearing an organic fluorophore attached at the periphery of the benzylic ring and an appropriate dark quencher located on the C-8 position of the guanine ring (Figure 1). Upon reaction with the SNAP-tag, the free quencher-bound guanine group is released into solution leading to a large increase in the relative fluorescence intensity of the fluorophore, which remains attached to the protein tag. Analysis of the structure of the wild-type human AGT suggested that the introduction of substituents at the C-8 position of guanine would have few sterically unfavorable interactions within the active site of the protein.[33] Several other positions were ruled out based on previous studies showing that minor changes such as the addition of methyl groups to the N-2 or N-7 position of *O*^6^-benzylguanine impedes AGT activity,[34] while caging of BG substrates at N-7 or N-9 abolishes activity towards the SNAP-tag.[35] It is important to note that although the presence of substituents at solvent-exposed C-8 and N-9 positions may be tolerated for wild-type AGT, N-9 substituted BG substrates exhibit much lower reactivity towards the SNAP-tag because mutations have been introduced in the protein that obstruct the guanine-binding pocket at the N-9 position.[36] These observations have been confirmed by Komatsu et al., who measured the activity of the SNAP-tag protein with various C-8 and N-9 BG derivatives. They found that while the C-8 modification decreased the labeling rate by a factor of 4 (C-8-carboxyethyl-BG, *k*∼5×10^3^ s^−1^ m^−1^) compared to unmodified BG substrates (BG, *k*∼2×10^4^ s^−1^ m^−1^), the N-9 modification dramatically reduced the SNAP-tag labeling rate (N-9-methoxyethanol-BG, *k*<1 s^−1^ m^−1^).[23]

**Scheme 1:**
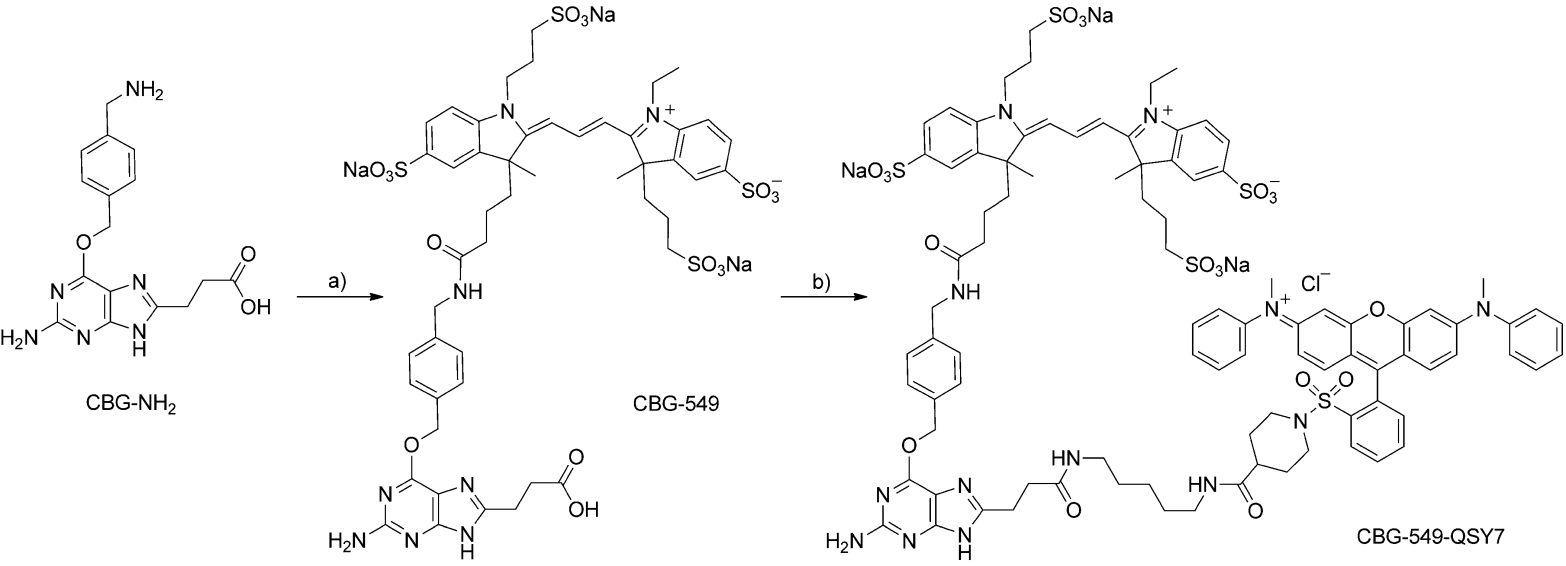
Synthesis of the SNAP-tag fluorogenic probe CBG-549-QSY7. a) DY-549 NHS, triethylamine, DMF, RT; b) QSY-7 amine, HBTU, triethylamine, 1 h, RT.

The efficiency of FRET-based quenching is dependent on the distance between the fluorophore donor and the quencher acceptor, and the degree of overlap between the fluorophore emission and quencher absorption spectra. Therefore, we selected fluorophore/quencher pairs from commercially available probes displaying significant spectral overlap and complementary reactive chemical functionalities for the design of the fluorogenic substrates (Table 1). We also employed a broad range non-fluorescent quencher, IRDye QC-1, which has been reported to efficiently quench the visible to near-infrared emission of fluorophores.[37] Substrates were prepared in a sequential one-pot, 2-step protocol, starting from the coupling of the CBG-NH_2_ building block with succinimidyl esters of the corresponding fluorophores, followed by a HBTU-mediated reaction with the amino-modified quenchers (Scheme 1). The synthetic strategy was designed to avoid the need for purification of any intermediate compounds and to expedite the assembly of fluorophore/quencher pairs into a SNAP-tag-reactive benzylguanine core for initial screening studies.

**Table 1:** Characterization of fluorogenic SNAP-tag substrates

Substrate	Quenching efficiency[a] [%]	*t*_1/2_ [s]	Kinetics (SNAP_f_)[b]*k* [m^−1^ s^−1^]	rel. rate
SNAP-Surface 488	80.4±2.2	11±1	12 183±823	80
CBG-488-DABCYL	94.6±0.7	905±272	162±42	1.1
CBG-488-TQ2	98.7±0.2	213±106	831±549	5.5
SNAP-Surface-549	55.3±4.7	13±2	11 138±1829	73
CBG-549-TQ3	97.0±0.1	239±71	616±188	4.0
CBG-549-QSY7	98.4±0.1	1027±457	152±58	1.0
CBG-549-QC1	84.3±0.3	n.d.	n.d.	n.d.
CBG-TF3	18.5±2.1	29±11	5213±1837	34
CBG-TF3-DABCYL	97.4±0.1	596±310	310±226	2.0
CBG-TF3-TQ3	95.9±0.7	362±189	479±285	3.2
SNAP-Surface AF647	7.3±1.4	34±18	4768±2093	31
CBG-AF647-QC1	95.8±0.1	499±106	286±61	1.9
SNAP-Surface 647	22.4±0.8	n.d.	n.d.	n.d.
CBG-647-QC1	85.5±0.6	n.d.	n.d.	n.d.
CBG-TF5	−18.9±2.4	34±9	4492±1075	30
CBG-TF5-QSY21	91.6±0.5	498±90	284±48	1.9
CBG-TF5-QXL670	76.5±0.6	n.d.	n.d.	n.d.

[a] Plate assay average of triplicate

[b] In-gel assay average of triplicate

n.d.: not determined.

The fluorogenic probes CBG-488-DABCYL and CBG-488-TQ2 were synthesized using the fluorescent dye ATTO 488, which has an emission maximum at 523 nm, and the dark quenchers DABCYL and TQ2, which exhibit absorption maxima between 450 and 550 nm. The fluorogenic probes CBG-549-TQ3, CBG-549-QSY7 and CBG-TF3-TQ3 were synthesized using the fluorescent dyes DY-549 and TF3, which have emission maxima at 575 and 578 nm, respectively, and the quenchers TQ3 and QSY-7, which exhibit absorption maxima between 550 and 600 nm. The fluorogenic probes CBG-TF5-QSY21 and CBG-TF5-QXL670 were synthesized using the fluorescent dye TF5, which has emission maximum at 670 nm, and the quenchers QSY-21 and QXL-670, which display absorption spectra ranging from 600–700 nm. The fluorogenic probes CBG-549-QC1, CBG-647-QC1 and CBG-AF647-QC1 were synthesized using the fluorescent dyes DY-549, DY-647, and Alexa Fluor 647, which have emission maxima at 575, 672, and 665 nm, respectively, and the non-fluorescent broad range quencher dye IRDye QC-1, which is compatible with fluorophores that emit in the 500–800 nm range. The final products were purified by reverse-phase C18 HPLC and characterized by HRMS and UV spectroscopy. The fluorescent covalent adducts resulting from the reaction between the SNAP-tagged protein and the fluorogenic substrates were further characterized by mass spectrometry (Table S2 in the Supporting Information). The synthetic approach is illustrated for the CBG-549-QSY7 substrate (Scheme 1).

### Characterization of a fast-labeling SNAP-tag variant

SNAP_f_, a SNAP-tag variant based on a previously described hAGT mutant,[38] was used for the labeling experiments. The additional point mutations in SNAP_f_ are described in Figure S1. SNAP_f_ carries 19 amino acid substitutions and a C-terminal deletion compared to wild-type AGT, and ten extra mutations compared to SNAP26m, which until 2011 was the commercially available version of SNAP-tag from New England Biolabs. We first compared the reactivity of purified SNAP_f_ and SNAP26m proteins towards several fluorophore BG conjugates using their second order rate constants. SNAP_f_ showed up to tenfold increased in vitro activity relative to SNAP26m (Table S1). The time required for 50 % labeling of SNAP_f_ at 1 μm protein concentration with 5 μm SNAP-Surface 488 (BG-488), SNAP-Cell TMR-star, SNAP-Surface 549 (BG-549), and SNAP-Surface Alexa Fluor 647 (BG-AF647), was calculated to be 11, 12, 13, and 34 s, respectively.

Having shown that SNAP_f_ efficiently reacts with fluorescent BG substrates, we next examined the rate of reaction of SNAP_f_ with the self-quenching fluorogenic probes. An improved rate of covalent labeling was desirable as we expected the reactivity of substrates carrying a quencher group at the guanine C-8 position to be slower than C-8 unmodified BG substrates. This hypothesis was later validated by ourselves as well as Komatsu et al.[23] The fluorogenic probe CBG-488-TQ2 was used as a model to compare the relative activities of SNAP_f_ and SNAP26m. Initial experiments revealed that the labeling of SNAP_f_ with CBG-488-TQ2 was approximately tenfold faster than of SNAP26m (Figure S3). The SNAP_f_ mutant showed increased reactivity against various BG derivatives (Table S1) and proved to be essential to achieve experimentally useful reaction rates with the BG fluorogenic probes. Furthermore, we found that a single specific mutation (E30R) of SNAP_f_ transferred to the benzylcytosine-specific CLIP-tag resulted in increased labeling rates of this mutant (CLIP_f_) towards CLIP-tag substrates (data not shown).

### In vitro characterization of fluorogenic substrates

Based on these results, we decided to investigate the labeling of SNAP_f_ with a collection of fluorogenic substrates containing combinations of fluorophore/quencher pairs spanning the visible spectrum. We first determined the quenching efficiency of each substrate. To this end, substrate (5 μm) was incubated in the presence or absence of purified SNAP_f_ protein (10 μm), and the fluorescence recovery was monitored in 5 min intervals over 2 h at 25 °C using a scanning fluorometer. In vitro quenching assays indicated 76–99 % fluorescence recovery after incubation of the fluorogenic probes with SNAP_f_ (Table 1). The majority of quenchers were effective, resulting in substantially lower fluorescence signals for the free substrate species compared to unquenched BG conjugates. No single fluorophore or quencher was universally better than the others. Rather, our results indicate that quenching and labeling efficiencies were highly dependent on the pairwise combinations.

Several fluorogenic substrates showed quenching efficiencies greater than 95 %, which corresponds to a ∼20-fold increase in the fluorescence signal upon labeling the SNAP_f_ protein. CBG-488-TQ2 and CBG-549-QSY7 showed quenching efficiencies greater than 98 % or ∼50-fold increase in the fluorescence signal. It has previously been observed that guanine can quench the fluorescence of particular dyes by photo-induced electron transfer (PET).[39] Stohr et al. investigated 21 different BG-fluorophore conjugates, some of which showed a tenfold increase in fluorescence emission upon reaction with SNAP-tag. Assessment of our BG-fluorophores indicate that guanine-induced PET quenches the following fluorophores to various degrees: SNAP-Surface 488 (80 %), SNAP-Surface 549 (55 %), SNAP-Surface 647 (22 %), and SNAP-Surface Alexa Fluor 647 (7 %). Therefore, we conclude that the overall observed quenching efficiency of the fluorogenic substrates is a result of both the FRET-based and the guanine PET-based quenching.

**Figure 2:**
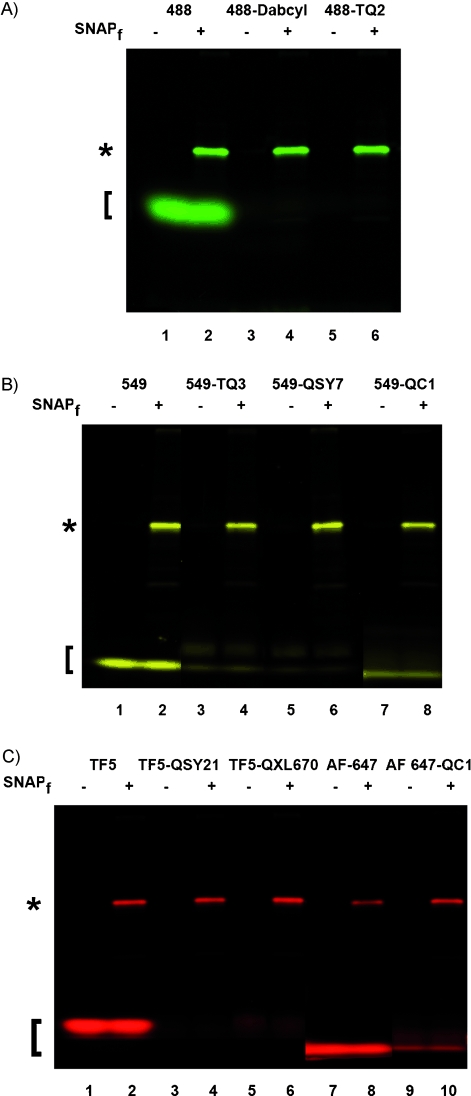
In-gel detection analysis of fluorogenic substrates. Purified SNAP_f_ protein (+) was labeled by various fluorogenic substrates: SNAP-Surface 488 (BG-488), CBG-488-Dabcyl, CBG-488-TQ2, SNAP-Surface 549 (BG-549), CBG-549-TQ3, CBG-549-QSY7, CBG-549-QC1, CBG-TF5, CBG-TF5-QSY21, CBG-TF5-QXL670, SNAP-Surface Alexa Fluor 647 (BG-AF647), and CBG-AF647-QC1. The samples were subjected to SDS-PAGE and scanned with a Typhoon 9400 imager using appropriate filter sets. The upper band (marked with *) corresponds to the labeled SNAP_f_ protein; lower bands correspond to unreacted dyes.

We also analyzed quenching and labeling efficiencies by in-gel fluorescence scanning (Figure 2). For this purpose, substrate (10 μm) was incubated in the presence or absence of purified SNAP_f_ protein (5 μm) for 30 min, followed by SDS-PAGE and analysis with a fluorescence imager. In-gel detection analysis indicated the SNAP_f_ protein was labeled with fluorogenic substrates (Figure 2 A lanes 4 and 6, Figure 2 B lanes 4, 6, and 8, and Figure 2 C lanes 4, 6, and 10) with an intensity that was comparable to the SNAP_f_ product labeled with unquenched substrates (Figure 2 A lane 2, B lane 2, and C lanes 2 and 8). Moreover, we observed a significant reduction in the fluorescence intensity of the unreacted fluorogenic substrates (lower bands; Figure 2 A lanes 3–6, B lanes 3–8, and C lanes 3–6 and 9–10) compared to the unquenched substrates (lower bands; Figure 2 A lanes 1–2, B lanes 1–2, and C lanes 1–2 and 7–8). As expected, these results clearly demonstrate an efficient fluorescence recovery after incubation of the quencher-containing substrates with the SNAP_f_ protein.

### Kinetic analysis

A kinetic analysis of the SNAP labeling reaction was carried out with the fluorogenic substrates having the highest quenching efficiencies. The kinetic analysis was determined by incubating substrate (5 μm) with purified SNAP_f_ protein (1 μm) in reaction buffer (1 mm DTT, 1×PBS) at 22 °C and removing aliquots at 0, 0.25, 0.5, 0.75, 1, 2, 4, 8, 16, 32, and 64 min. Labeling efficiency was evaluated using SDS-PAGE and in-gel fluorescence scanning. The individual rate constants were determined from the average of triplicate experiments (Table 1). Two important results emerged from these kinetics studies. First, the quencher-containing substrates showed a marked decrease in the second order rate constants compared to the corresponding unquenched substrates. We attribute this decrease to an adverse steric effect caused by the incorporation of a quencher at the C-8 position of guanine on binding and transfer of the fluorophore moiety to the protein tag. This is supported by the observation that incorporation of different quenchers reduced the reactivity of the fluorogenic substrates from 10- to 100-fold relative to their parent substrates. For instance, CBG-488-TQ2 reacts 15-fold slower than SNAP-Surface 488, whereas CBG-488-DABCYL reacts about 72-fold slower than SNAP-Surface 488. Despite the fact that fluorogenic substrates are significantly less reactive (*t*_1/2_ 3 to 18 min) than the substrates without a quencher moiety (*t*_1/2_ 11 to 34 s), ESI-TOF mass analysis indicates complete labeling of SNAP_f_ after 60 min incubation with most of the substrates (Table S2). Additionally, our results show the fluorophore itself affects activity towards SNAP-tag. For example, SNAP-Surface 488 and SNAP-Surface 549 react up to threefold faster than SNAP-Surface Alexa Fluor 647. Taken together, these data suggest both fluorophore and quencher affect the binding and conjugation of the substrate to the SNAP_f_ protein.

### Quantification of fusion proteins in cell lysates

**Figure 3:**
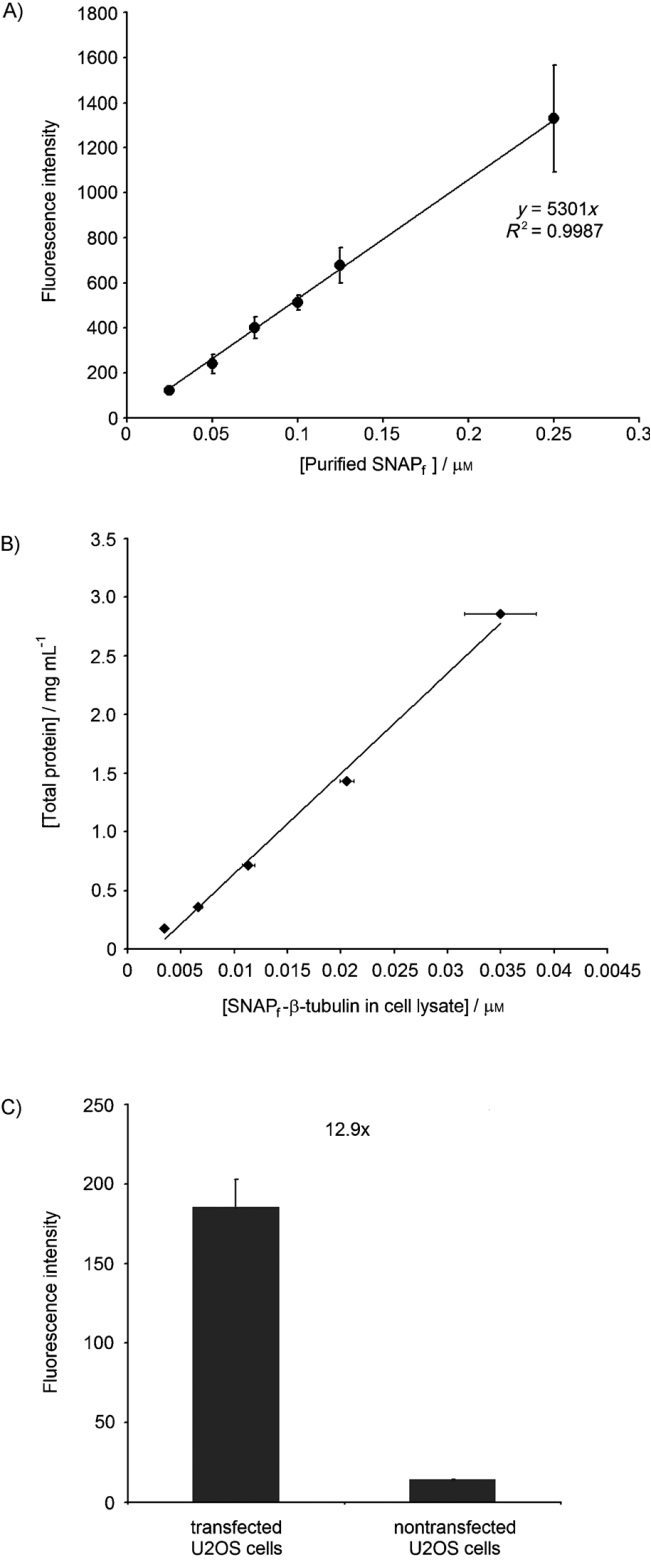
Quantification of SNAP_f_-β-tubulin in U2OS cell lysates. A) Standard curve for determining SNAP_f_ protein concentration. Purified SNAP_f_ protein at various concentrations (0.025, 0.05, 0.075, 0.1, 0.125, and 0.25 μm) was incubated with CBG-488-TQ2 (0.5 μm) and nontransfected U2OS cell lysates at room temperature for 4.5 h. Results are representative of experiments performed in triplicate. Fluorescence intensity was recorded at 526 nm (emission maximum) upon excitation at 488 nm. B) SNAP_f_-β-tubulin concentration. Cells stably expressing SNAP_f_-β-tubulin were lysed, diluted with PBS (1:1, 1:2, 1:4, 1:8, and 1:16), and treated with CBG-488-TQ2 (0.5 μm). The measured fluorescence intensities were converted into SNAP_f_-β-tubulin protein concentrations by using the standard curve generated for the SNAP_f_ protein. C) Fluorescence intensity of cell lysates from U2OS cells stably expressing SNAP_f_-β-tubulin and from nontransfected U2OS cells incubated with CBG-488-TQ2 (0.5 μm).

We next investigated the application of these fluorogenic substrates as a tool for protein quantification. In view of the fact that the fluorescence recovery of the fluorogenic substrates directly correlates with their labeling by SNAP_f_, we hypothesized that their relative fluorescence intensity could serve as the basis for the measurement of the concentration of a given tagged protein. To test this hypothesis, the fluorescence intensity of CBG-488-TQ2 after incubation with various concentrations of purified SNAP_f_ protein was measured. All reactions were carried out in triplicate and a nontransfected U2OS cell lysate was included in the reaction buffer to mimic mammalian cell lysis conditions. The results show a linear correlation (*R*>0.99) between the fluorescence signal and SNAP_f_ protein concentration (Figure 3 A).

Next the assay was extended to a U2OS stable cell line expressing SNAP_f_-β-tubulin. Serial dilution of the total U2OS cell lysate was incubated with 0.5 μm of CBG-488-TQ2 for 4.5 h at room temperature, and the total protein concentration (mg mL^−1^) was plotted against the concentration of SNAP_f_-β-tubulin in the cell lysate (Figure 3 B). We observed a reproducible linear relationship between the SNAP_f_-β-tubulin protein concentration and the total protein concentration, and that the signal-to-background ratio over the nontransfected cell lysate was approximately 13:1 (Figure 3 C). To test whether this assay was compatible with a high-throughput screening format, we plated the U2OS cells stably expressing SNAP_f_-β-tubulin into a 96-well plate, directly lysed cells by adding lysis buffer to the wells, and then detected the fluorescence recovery of the CBG-488-TQ2 substrate under the same conditions. In these experiments, the signal-to-noise ratio (average fluorescence intensity/standard deviation) and the signal-to-background ratio (average fluorescence intensity of transfected U2OS cells/average fluorescence intensity of nontransfected U2OS cells) were 316.8 and 5.3, respectively, indicating the versatility and robustness of the fluorogenic substrates and their potential for direct detection and quantification of tagged proteins in complex biosystems.

### Wash-free labeling of fusion proteins in living cells

**Figure 4:**
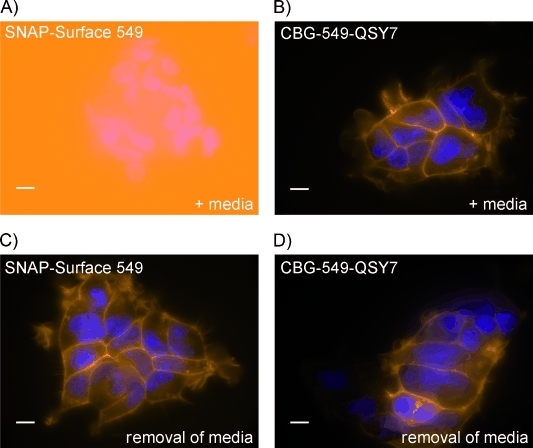
Comparison of SNAP-Surface 549 and CBG-549-QSY7 substrates for labeling SNAP_f_-EGFR in living cells. Live HEK 293 cells stably expressing SNAP_f_-EGFR were incubated for 30 min at 37 °C with A) SNAP-Surface 549 (1 μm) or B) CBG-549-QSY7 (1 μm). SNAP-Surface Block was added to the cells (final concentration of 20 μm) to inhibit further labeling. Images C and D were obtained after replacing the labeling media with complete growth media containing SNAP-Surface Block (20 μm). Images were taken on a wide field Axiovert 200 m Zeiss microscope using a 63X objective and fixed exposure time (100 ms). Cells were counterstained with Hoechst 33342 for nuclei (blue). Scale bars: 10 μm.

Finally, we sought to demonstrate the feasibility of wash-free labeling of fusion proteins in living cells. To this end, we designed a SNAP_f_-EGFR fusion protein and stably expressed the fusion protein in HEK293 cells. EGFR was selected as a model system because it is thought to contribute to cell signaling[40, 41] and is implicated in many disease states.[42] To test if the labeling of the SNAP_f_-EGFR fusion protein could be performed in wash-free conditions, we selected the fluorogenic substrates that displayed quenching efficiencies greater than 90 % (i.e., at least tenfold increase in fluorescence signal upon SNAP_f_ labeling). Cells were incubated with 1 μm of each fluorogenic substrate for 30 min and then treated with SNAP-Surface Block (New England Biolabs) at a concentration of 20 μm to inhibit further labeling of SNAP_f_-EGFR. The fluorescence images of cells labeled with CBG-549-QSY7 revealed clear cell-surface imaging even in the presence of the labeling medium containing an excess of the unreacted fluorogenic substrate (Figure 4 B). Similarly, cells labeled with the fluorogenic substrate CBG-488-DABCYL, CBG-488-TQ2, CBG-AF647-QC1, and CBG-TF5-QSY21 showed high signal-to-background contrast both in the presence and in the absence of the labeling medium (Figures S4 B, S4 C, S5 B, and S6 B).

On the other hand, fluorescence images of the HEK293 cells labeled with conventional SNAP-tag substrates, such as SNAP-Surface 488, SNAP-Surface 549 and SNAP-Alexa Fluor 647, taken under the same conditions, showed cell surface staining with much less contrast and higher background signal (Figure 4 A and Figures S4 A, S5 A, and S6 A), therefore requiring a washing step to remove excess unreacted fluorophore. However, one should note that the cell images obtained with both unquenched and quenched substrates after rigorous washing are virtually indistinguishable. Notably, SNAP-Surface 488, whose fluorophore is strongly quenched by guanine, exhibited a remarkably high signal-to-noise ratio in live cells even before the removal of media and subsequent washing steps (Figure S4 A). It is clear from our observations that the data obtained for wash-free live cell labeling is consistent with the high quenching efficiencies observed in the in vitro assays. Furthermore, distinct and specific surface labeling of HEK293 expressing SNAP_f_-EGFR could be visualized after an incubation time as short as 5 min using 5 μm CBG-549-QSY7. This demonstrates that the visualization of cell membrane-localized targets can be achieved within a few minutes of labeling without the removal of any unreacted substrates.

Next, we investigated whether the fluorogenic substrates could be used for in vivo labeling of intracellular targets. We found that all fluorogenic probes described in the Table 1 were cell-impermeant. These results are consistent with previous reports that fluorescent dyes carrying negatively charged groups cannot passively cross cell membranes.[43] Consequently, we evaluated some of the cytoplasmic delivery techniques that have been reported to allow membrane-impermeant fluorophore conjugates to be introduced into living cells.[43, 44] We successfully obtained wash-free images of intracellular SNAP_f_-tagged histone H2B or β-tubulin in U2OS cells using a bead-loading method to deliver the CBG-549-TQ3 probe (Figure S7 A and B). In addition, commonly used transfection reagents, such as Fugene 6 (Roche), were also shown to deliver cell-impermeant substrates and thus enable the labeling of cytosolic SNAP-tagged proteins (Figure S7 C). It should be noted that only a small fraction (<5 %) of the cells were labeled using either the glass beads or transfection reagents.

**Figure 5:**
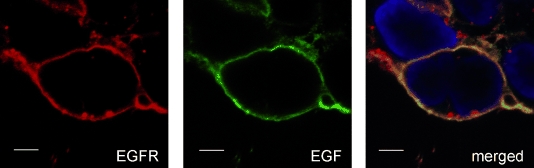
Live cell imaging of colocalization of SNAP_f_-EGFR and EGF-CLIP_f_. HEK293 cells stably expressing SNAP_f_-EGFR were labeled with 5 μm CBG-549-QSY7 (red) at 25 °C for 5 min. Cells were then incubated for 2 min with EGF-CLIP_f_ labeled with CLIP-Surface 488 (green) at 500 ng mL^−1^ prior to imaging by confocal fluorescence microscopy. Nuclear staining was performed with Hoeschst 33342 (blue). Right side panel shows the merged micrographs of the FITC, rhodamine and DAPI channels. Scale bars: 10 μm.

Lastly, we demonstrated the application of this approach for a two-color fluorescence visualization of the EGF/EGFR ligand–receptor complex (Figure 5). To study the colocalization of the ligand–receptor complex, EGF was cloned as a fusion to the engineered CLIP_f_ mutant. CLIP_f_ contains an extra amino acid substitution (E30R) compared to the CLIP-tag. For the colocalization assay, purified recombinant EGF-CLIP_f_ was fluorescently labeled with CLIP-Surface 488. HEK293 cells stably expressing SNAP_f_-EGFR were labeled with CBG-549-QSY7. Cells were then incubated for 2 min with labeled EGF-CLIP_f_ and directly imaged by a confocal microscope without removal of the medium or any washing step. Analyses of fluorescence images clearly showed colocalization (yellow) of labeled SNAP_f_-EGFR (red) and its receptor EGF-CLIP_f_ (green). The data demonstrate the potential of this system for rapid and sensitive detection of EGFR and the EGFR/EGF receptor–ligand complex. The possibility of performing real-time analysis of receptor endocytosis in response to extracellular stimulus, such as ligand binding, could now be envisaged as a prospective application of this system.

## Conclusions

In summary, we have developed and characterized novel fluorogenic substrates of high quenching efficiency for analysis of dynamic processes in living cells utilizing a new generation of fast self-labeling SNAP-tag protein. This site-specific labeling system offers unique advantages, including wash-free, real-time visualization of SNAP-tagged proteins in cell lysates and in living cells. This method should be particularly applicable in systems where sensitive detection is required, including protein quantification and single-molecule microscopy, or in high-throughput screening platforms where the response of screening assays needs to be clearly defined and assessed in a timely fashion, minimizing the interference from nonspecific fluorescent species and avoiding separation steps which can make automation difficult. The labeling system described here opens new avenues for the spatiotemporal resolution of fluorescence signals that is required for real-time monitoring of highly dynamic processes in living cells, and for high-throughput screening of proteins in complex biosystems and drug discovery.

## Experimental Section

**Chemical methods:** Commercially available compounds were used without further purification. All fluorogenic substrates for the labeling of SNAP-tag fusion proteins were prepared by reacting the building block CBG-NH_2_ (New England Biolabs) with commercially available *N*-hydroxysuccinimide esters of the corresponding fluorophores and amines of the corresponding quenchers. ATTO-488 NHS was purchased from ATTO-TEC GmbH (Siegen, Germany). Tide Fluor 3 (TF3) NHS, Tide Fluor 5 (TF5) NHS, Tide Quencher 2 (TQ2) acid, Tide Quencher 3 (TQ3) acid were purchased from AAT Bioquest, Inc. (Sunnyvale, CA). Dabcyl C2 amine and QXL670 C2 amine were purchased from AnaSpec, Inc. (Fremont, CA). DY-549 NHS and DY-647 NHS were purchased from Dyomics GmbH (Jena, Germany). Alexa Fluor 647 NHS, QSY-7 amine, and QSY-21 NHS were purchased from Life Technologies Co. (Carlsbad, CA). IRDye QC-1 NHS was provided by LI-COR Biosciences (Lincoln, NE). QSY-21 amine, TQ2 amine, TQ3 amine, and IRDye QC-1 amine were synthesized by reacting *N*-Fmoc-1,2-diaminoethane hydrobromide (Sigma–Aldrich) with commercially available QSY-21 NHS, TQ2 acid, TQ3 acid, and IRDye QC-1 NHS, respectively. Due to the confidential or proprietary nature of the majority of fluorophores and quenchers used in this study, very limited information about chemical structures is available from dye manufacturers.

**Purification and analysis of substrates:** Reversed-phase high-performance liquid chromatography (RP-HPLC) was performed on an Agilent LCMS Single Quad System 1200 Series (analytical) and Agilent 1100 Preparative-scale Purification System (semi-preparative). Analytical HPLC was performed on a Waters Atlantis T3 C18 column (2.1×150 mm, 5 μm particle size) at a flow rate of 0.5 mL min^−1^ with a binary gradient from solvent A (0.1 % aq. formic acid) to solvent B (acetonitrile with 0.1 % formic acid) and monitored by UV–visible absorbance at 280 nm and at the absorption maximum of each fluorophore. Semi-preparative HPLC was performed on a VYDAC 218TP series C18 polymeric reversed-phase column (22×250 mm, 10 μm particle size) at a flow rate of 20 mL min^−1^ using a water/acetonitrile gradient with trifluoroacetic acid (0.1 %) or 1 m triethyl ammonium bicarbonate buffer (0.1 %). Mass spectra were recorded by electrospray ionization (ESI) on Agilent 6210 Time-of-Flight (TOF) LC/MS System. UV spectra were recorded on a Beckman DU 640B Spectrophotometer.

**Synthesis of fluorogenic substrates:** Reactions (1–2 μmol scale) were performed at room temperature in *N,N*-dimethylformamide in the presence of CBG-NH_2_ (1.0 equiv), triethylamine (2.0 equiv), and the fluorophore N-hydroxysuccinimidyl ester (1.0 equiv). The mixture was stirred for 12 h. Then the corresponding quencher amine (1.1 equiv), HBTU (1.5 equiv), and triethylamine (2.0 equiv) were added. The reaction completion was monitored by LCMS. Typically, after 1 h stirring, the mixture was concentrated, purified by RP-HPLC and lyophilized. Each substrate was analyzed by high-resolution mass spectrometry and UV absorption. Isolated yields are given in parentheses and are not optimized. The following substrates were purified using a water/acetonitrile gradient: SNAP-Surface 488 (70 %): ESI-TOFMS *m/z* 842.2027 [*M*+H]^+^ (calcd for C_38_H_35_N_9_O_10_S_2_, *m/z* 842.2021); UV (pH 7.5) *λ*_max_=507 nm. CBG-488-DABCYL (32 %): ESI-TOFMS *m/z* 1207.3854 [*M*+H]^+^ (calcd for C_58_H_58_N_14_O_12_S_2_, *m/z* 1207.3873); UV (pH 7.5) *λ*_max_=505 nm. CBG-488-TQ2 (28 %): ESI-TOFMS *m/z* 1320.4156 [*M*+H]^+^ (calcd for C_63_H_65_N_15_O_12_S_3_, *m/z* 1320.4172); UV (pH 7.5) *λ*_max_=503 nm. SNAP-Surface 549 (76 %): ESI-TOFMS *m/z* 1069.2551 [*M*−H]^−^ (calcd for C_46_H_54_N_8_O_14_S_4_, *m/z* 1069.2570); UV (H_2_O) *λ*_max_=555 nm. CBG-TF3 (51 %): ESI-TOFMS *m/z* 783.3248 [*M*+H]^+^ (calcd for C_43_H_42_N_8_O_7_, *m/z* 783.3249); UV (MeOH) *λ*_max_=545 nm. CBG-TF3-DABCYL (21 %): ESI-TOFMS *m/z* 1076.4851 [*M*+H]^+^ (calcd for C_60_H_61_N_13_O_7_, *m/z* 1076.4890); UV (MeOH) *λ*_max_=556 nm. SNAP-Surface 647 (68 %): ESI-TOFMS *m/z* 895.3237 [*M*+H]^+^ (calcd for C_45_H_50_N_8_O_8_S_2_, *m/z* 895.3266); UV (EtOH) *λ*_max_=652 nm. CBG-TF5 (63 %): ESI-TOFMS *m/z* 1203.2896 [*M*+H]^+^ (calcd for C_54_H_58_N_8_O_16_S_4_, *m/z* 1203.2926); UV (MeOH) *λ*_max_=655 nm. The following substrates were purified using a water/acetonitrile gradient with trifluoroacetic acid (0.1 %): CBG-549-TQ3 (13 %): ESI-TOFMS *m/z* 808.7301 [*M*−2H]^2−^ (calcd for C_73_H_85_N_15_O_18_S_5_, *m/z* 808.7328); UV (MeOH) *λ*_max_=558 nm. CBG-549-QSY7 (54 %): ESI-TOFMS *m/z* 933.8112 [*M*+2H]^2+^ (calcd for C_93_H_103_N_13_O_19_S_5_, *m/z* 933.8127); UV (MeOH) *λ*_max_=559 nm. CBG-TF3-TQ3 (17 %): ESI-TOFMS *m/z* 1260.5157 [*M*+H]^+^ (calcd for C_67_H_69_N_15_O_9_S, *m/z* 1260.5196); UV (MeOH) *λ*_max_=556 nm. SNAP-Surface Alexa Fluor 647 (87 %): ESI-TOFMS *m/z* 1111.2993 [*M*+H]^+^ (calcd for C_49_H_58_N_8_O_14_S_4_, *m/z* 1111.3028); UV (MeOH) *λ*_max_=651 nm. CBG-TF5-QXL670 (47 %): ESI-TOFMS *m/z* 925.8117 [*M*+2H]^2+^; UV (MeOH) *λ*_max_=657 nm. CBG-TF5-QSY21 (67 %): ESI-TOFMS *m/z* 954.7859 [*M*+2H]^2+^ (calcd for C_97_H_98_N_13_O_19_S_5_, *m/z* 954.7887); UV (MeOH) *λ*_max_=656 nm. The following substrates were purified using water/acetonitrile gradient with 1 m triethylammonium bicarbonate buffer (0.1 %): CBG-549-QC1 (11 %): ESI-TOFMS *m/z* 1121.7978 [*M*+2H]^2−^ (calcd for C_101_H_125_ClN_12_O_28_S_8_, *m/z* 1121.8057); UV (EtOH) *λ*_max_=561 nm. CBG-AF647-QC1 (22 %): ESI-TOFMS *m/z* 1141.8104 [*M*−2H]^2−^ (calcd for C_103_H_128_ClN_13_O_28_S_8_, *m/z* 1141.8150); UV (MeOH) *λ*_max_=651 nm. CBG-647-QC1 (31 %): ESI-TOFMS *m/z* 1035.3349 [*M*+H]^2+^ (calcd for C_99_H_120_ClN_13_O_22_S_6_, *m/z* 1035.3376); UV (EtOH) *λ*_max_=653 nm. Detailed experimental protocol and ^1^H NMR spectrum for CBG-549-QSY7 (Scheme S1) can be found in the Supporting Information. Substrates were further characterized by ESI-TOF mass spectrometry after their binding to the SNAP_f_ protein (Table S2).

**Expression constructs** (Figure S2): pSNAP_f_ was constructed by insertion of the cDNA encoding SNAP_f_, synthesized by IDT, between the restriction sites EcoRI and SbfI of pSNAP-tag(m) (New England Biolabs). This SNAP-tag variant, SNAP_f_, contains 19 amino acid substitutions and an additional 24-residue deletion at the C-terminus compared to the wild-type AGT. Constitutive expression of the SNAP_f_ is under the control of a CMV promoter. The cDNA encoding the CLIP_f_ was introduced between the EcoRI and SbfI sites of pSNAP_f_, resulting in pCLIP_f_. pSNAP_f_-tag(T7) and pCLIP_f_-tag(T7) were constructed by replacing the SNAP-26 m coding region of pSNAP-tag(T7)-2 using the unique EcoRI and SbfI sites with the coding regions of SNAP_f_ and CLIP_f_, respectively.

The mouse EGF coding sequence was fused in-frame to the 5′-end of SNAP_f_ and CLIP_f_, and a hexahistidine tag (His_6_) was fused to the 3′-end of SNAP_f_ and CLIP_f_ in pSNAP_f_-tag(T7) and pCLIP_f_-tag(T7), respectively. The resulting plasmids pEGF-SNAP_f_-His_6_ and pEGF-CLIP_f_-His_6_ were used for expression of EGF-SNAP_f_ and EGF-CLIP_f_ fusion proteins in *E. coli* and subsequent affinity purification by Ni-NTA agarose (Qiagen). A linker encoding the signal sequence of EGFR, formed by annealing 5′-CTAGC ATGCG ACCCT CCGGG ACGGC CGGGG CAGCG CTCCT GGCGC TGCTG GCTGC GCTCT GCCCG GCGAG TCGGG CTG-3′- and 5′-AATTC AGCCC GACTC GCCGG GCAGA GCGCA GCCAG CAGCG CCAGG AGCGC TGCCC CGGCC GTCCC GGAGG GTCGC ATG-3′, was inserted into the 5′-MCS of the pSNAP_f_ vector using the unique NheI and EcoRI sites (underlined). Subsequently the coding sequence of mature EGFR (GeneCopoeia) was amplified by PCR and subcloned into the plasmid described above using the unique SbfI and NotI sites, creating pSNAP_f_-EGFR. SNAP_f_-β-tubulin was generated from the human β-tubulin coding sequence (Open Biosystems) which was amplified by PCR and fused in-frame to the 5′-end of SNAP_f_ in the pSNAP_f_ vector.

**Fluorescence in-gel detection:** SNAP_f_ protein was labeled at 37 °C for 30 min in the presence of SNAP_f_ (5 μm), BG conjugate (10 μm) and DTT (1 mm) in PBS. The samples were submitted to electrophoresis on a 10–20 % Tris-glycine gel under denaturing conditions. The gels were scanned using a Typhoon 9400 imager at 300 V PMT with a 488/526 nm (Figure 2 A, 488 in green), 532/580 nm (Figure 2 B, 549 in orange) or 633/670 nm excitation/emission filter set (Figure 2 C, TF5 and Alexa Fluor 647 in red).

**Assay of quenching efficiency:** Fluorescence signals of the SNAP_f_ proteins labeled with a fluorophore from a quenched or non-quenched substrate were analyzed with a FLEXstation scanning fluorometer (Molecular Devices). The reactions were performed in 96-well plates (Costar) and the fluorescence was measured at the appropriate wavelength. Reactions were carried out with dye (5 μm) and DTT (1 mm) in PBS in the presence or absence of SNAP_f_ protein (10 μm). SNAP-Surface 488 and its fluorogenic derivatives were excited at 488 nm and measured at the maximum emission wavelength of 526 nm. SNAP-Surface 549, CBG-TF3 and their fluorogenic derivatives were excited at 546 nm and measured at the maximum emission wavelength of 580 nm. Fluorescence of SNAP-Surface 647, SNAP-Alexa Fluor 647, CBG-TF5 and their fluorogenic derivatives was read at 636 nm with maximum emission of 670 nm. Fluorescence was followed in 5 min intervals over 2 h at 25 °C. Quenching efficiencies were calculated by the equation *E*=1−(*I*_FD_/*I*_SNAPf_), where *I*_FD_ indicates fluorescence intensity of free dyes and *I*_SNAPf_ indicates fluorescence intensity of labeled SNAP_f_ protein at the end of the 2 h reaction.

**Kinetic study:** Labeling reactions were carried out at 22 °C in the presence of dye (5 μm), SNAP_f_ protein (1 μm) and DTT (1 mm) in PBS. At each of the following time points: 0, 15, 30 or 45 s, 1, 2, 4, 8, 16, 32 or 64 min, 18 μL of the labeling reaction was removed and added to a microfuge tube containing 18 μL of 3×Red SDS-PAGE loading buffer (New England Biolabs). After boiling the samples for 5 min, each sample (7.5 μL) was loaded on a 10–20 % Tris-glycine gel (Invitrogen). Following separation of proteins and free dyes on SDS-PAGE, the labeled SNAP_f_ protein was detected with fluorescence imager Typhoon 9400 (GE Healthcare). Gel scanning was performed with appropriate filter sets: excitation at 488 nm and emission at 526 nm for SNAP-Surface 488 and its fluorogenic derivatives; excitation at 533 nm and emission at 580 nm for SNAP-Surface 549, CBG-TF3 and their fluorogenic derivatives; excitation at 633 nm and emission at 670 nm for SNAP-Surface 647, SNAP-Alexa Fluor 647, CBG-TF5 and their fluorogenic derivatives. The imaging data were quantified with ImageQuant TL software (GE Healthcare). The data were fitted to an exponential rise model using the KaleidaGraph 4.0 software (Synergy Software) to get the pseudo-first-order rate constants. Second-order rate constants were then obtained by dividing the pseudo first-order constant by the concentration of substrate.

**Quantification of SNAP_f_-β-tubulin in cell lysates:** To generate a standard curve of fluorescence intensity versus SNAP_f_ protein concentration, purified SNAP_f_ protein (25 μL) at a final concentration of 0.025, 0.05, 0.075, 0.1, 0.125, and 0.25 μm were incubated with CBG-488-TQ2 (2 μm, 25 μL, final concentration 0.5 μm) and of cell lysate (50 μL) from nontransfected U2OS cells at room temperature for 4.5 h. The reaction was performed in triplicate in a 96-well plate (Costar). The fluorescence intensity was recorded at 526 nm emission maximum upon excitation at 488 nm and plotted against SNAP_f_ protein concentration. The curve was fitted to a linear equation.

The concentration of SNAP_f_-β-tubulin was measured from cell lysates of U2OS cells stably expressing SNAP_f_-β-tubulin. Cells grown at 37 °C in phenol red-free DMEM medium supplemented with 10 % fetal bovine serum (FBS), L-glutamine (2 mm), penicillin (100 units per mL), streptomycin (100 μg mL^−1^) and G418 (200 μg mL^−1^) were harvested from a 75 cm^2^; cell culture flask (BD Falcon) with 0.25 % trypsin treatment, then washed and spun down. The cell pellet was lysed in 500 μL of CelLytic M cell lysis reagent (Sigma–Aldrich) for 15 min at room temperature. Total protein concentration was determined by the Bradford assay. The cell lysate was serially diluted with PBS buffer (1:1, 1:2, 1:4, 1:8, and 1:16) to generate cell lysate samples with various total protein concentrations. 50 μL of each dilution was mixed with 1 μm CBG-488-TQ2 (50 μL, final concentration 0.5 μm) and incubated at room temperature for 4.5 h. The reaction was performed in triplicate in a 96-well plate and the fluorescence intensity was recorded at 526 nm upon excitation at 488 nm. The fluorescence intensities were converted to SNAP_f_-β-tubulin protein concentrations by using the standard curve generated for SNAP_f_. The total protein concentration (mg mL^−1^) was plotted against the concentration of SNAP_f_-β-tubulin in the cell lysate (μm). The signal-to-noise (S/N) ratios were determined as S/N=(*I*_F_−*I*_B_)/SD, where *I*_F_ is the average fluorescence intensity, *I*_B_ is the average background intensity, and SD is the standard deviation of background. The signal-to-background (S/B) ratios were determined as S/B=*I*_FT_/*I*_FNT_, where *I*_FT_ is the average fluorescence intensity of transfected U2OS cells and *I*_FNT_ is the average fluorescence intensity of nontransfected U2OS cells.

**Live cell labeling and imaging:** Human embryonic kidney (HEK 293) cells stably transfected with pSNAP_f_-EGFR were maintained at 37 °C in phenol red-free DMEM medium supplemented with 10 % fetal bovine serum (FBS), penicillin (100 units per mL), streptomycin (100 μg mL^−1^) and G418 (200 μg mL^−1^). Cells were seeded in Lab Tek II chambered coverglasses (Nalge Nunc Int). At 24 h post-seeding, cell membrane-localized SNAP_f_-EGFR was labeled by incubation of live HEK 293 cells stably expressing SNAP_f_-EGFR with SNAP-tag substrate (1 μm) for 30 min at 37 °C. Then SNAP-Surface Block (New England Biolabs) was added to the cells (final concentration 20 μm) to inhibit further labeling. Images were taken on a wide-field Axiovert 200 m Zeiss microscope using a 63× objective and fixed exposure setting. Cell nuclei were counterstained with Hoechst 33342. For imaging with medium removal, labeling was carried out as above, except that labeling medium was replaced with complete growth medium containing SNAP-Surface Block (20 μm). Images were processed using AxioVision 4.7 software.

**EGF-CLIP_f_** **isolation and labeling:** Expression of recombinant EGF-CLIP_f_-His_6_ was performed in SHuffle T7 *E. coli* (New England Biolabs). EGF-CLIP_f_-His_6_ was purified from *E. coli* cell lysate using Ni-NTA Agarose (Qiagen). Analysis of protein expression and purification was done with Coomassie Blue-stained SDS-PAGE. Labeling of EGF-CLIP_f_-His_6_ was carried out with EGF-CLIP_f_-His_6_ (40 μm), CLIP-Surface 488 (15 μm) and DTT (1 mm) in PBS on ice for 4 h.

**Colocalization of SNAP_f_-EGFR and EGF-CLIP_f_:** HEK293 cells stably expressing SNAP_f_-EGFR were labeled with 5 μm CBG-549-QSY7 (red) at 25 °C for 5 min. Cells were then incubated for 2 min with EGF-CLIP_f_ labeled with CLIP-Surface 488 (green) at 500 ng mL^−1^ prior to imaging by confocal fluorescence microscopy. Cells were counterstained with Hoechst 33342 for nucleus (blue). Images were acquired on a Zeiss LSM 510 laser scanning confocal microscope using a 63X objective. Images were processed using LSM 510 Meta software.
