# Supplementary material for: Development of SNAP-Tag Fluorogenic Probes for Wash-Free Fluorescence Imaging
Source: Chembiochem. 2011 Jul 26;12(14):2217–26. doi: 10.1002/cbic.201100173 (PMC3213346; doi:10.1002/cbic.201100173)
Supplement: Supplementary file 1 [file cbic0012-2217-SD1.pdf]

CHEMBIOCHEM

Supporting Information

© Copyright Wiley-VCH Verlag GmbH & Co. KGaA, 69451 Weinheim, 2011

Development of SNAP-Tag Fluorogenic Probes for Wash-Free Fluorescence Imaging

Xiaoli Sun,[a] Aihua Zhang,[a] Brenda Baker,[a] Luo Sun,[a] Angela Howard,[a] John Buswell,[a] Damien Maurel,[b] Anastasiya Masharina,[b] Kai Johnsson,[b] Christopher J. Noren,[a] Ming-Qun Xu,*[a] and Ivan R. Corrêa, Jr.*[a]

cbic_201100173_sm_miscellaneous_information.pdf

```
AGT          MDKDCEMKRTTLDSPLGKLELSGCEQGLHEIKLLGKGTSA      40
SNAP26m      MDKDCEMKRTTLDSPLGKLELSGCEQGLHEIKLLGKGTSA      40
SNAPf        MDKDCEMKRTTLDSPLGKLELSGCEQGLHRIIFLGKGTSA      40

AGT          ADAVEVPAPAAVLGGPEPLMQCTAWLNAYFHQPEAIEEFP      80
SNAP26m      ADAVEVPAPAAVLGGPEPLMQATAWLNAYFHQPEAIEEFP      80
SNAPf        ADAVEVPAPAAVLGGPEPLMQATAWLNAYFHQPEAIEEFP      80

AGT          VPALHHPVFQQESFTRQVLWKLLKVVKFGEVISYQQLAAL     120
SNAP26m      VPALHHPVFQQESFTRQVLWKLLKVVKFGEVISYQQLAAL     120
SNAPf        VPALHHPVFQQESFTRQVLWKLLKVVKFGEVISYSHLAAL     120

AGT          AGNPKATRAVGGAMRGNPVPILIPCHRVVCSSGAVGNYSG     160
SNAP26m      AGNPAATAAVKTALSGNPVPILIPCHRVVSSSGAVGGYEG     160
SNAPf        AGNPAATAAVKTALSGNPVPILIPCHRVVQGDLDVGGYEG     160

AGT          GLAVKEWLLAHEGHRLGKPGLGGSSGLAGAWLKGAGATSG     200
SNAP26m      GLAVKEWLLAHEGHRLGKPGLG                       182
SNAPf        GLAVKEWLLAHEGHRLGKPGLG                       182

AGT          SPPAGRN                                      207
SNAP26m                                                   182
SNAPf                                                     182
```

Figure S1. Sequence alignment of wild type human repair protein O^6-alkylguanine-DNA alkyltransferase (hAGT), SNAP26m, and SNAP$_f$. The specific amino acid changes of the AGT mutants SNAP26m and SNAP$_f$ are depicted in boxes.

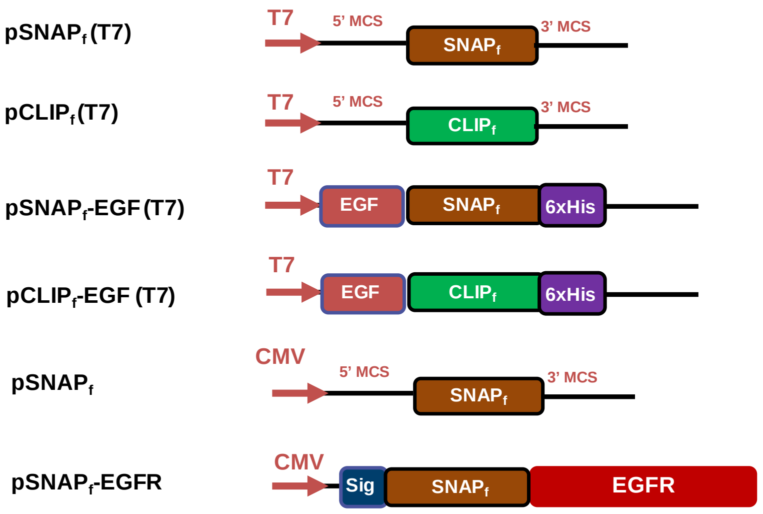

Figure S2. Schematic representation of SNAP$_f$ and CLIP$_f$ bacterial (T7) and mammalian constructs.

Table S1. Kinetics properties of $SNAP_f$ and SNAP26m[a]				
**Substrate**	**Mutant**	**t½[s]**	**k_{obs} [$M^{-1}s^{-1}$]**	**rel. rate**
SNAP-Surface 488	SNAP26m	88	1583	2.5
	$SNAP_f$	11	12183	19
SNAP-Cell TMR-Star	SNAP26m	90	1536	2.4
	$SNAP_f$	12	12033	19
SNAP-Surface 549	SNAP26m	129	1074	1.7
	$SNAP_f$	13	11138	18
SNAP-Surface Alexa Fluor 647	SNAP26m	219	634	1
	$SNAP_f$	34	4768	7.5

[a] Activities of $SNAP_f$ and SNAP26m mutants were measured using proteins purified from SNAP-intein-chitin binding domain (CBD) fusion constructs. The reactions were carried at 22°C using a 5:1 molar ratio of substrate to protein.

Table S2. Mass spectrometry characterization of labeled $SNAP_f$ protein[a]					
**Substrate**	**$SNAP_f$+Dye Expected**	**$SNAP_f$+Dye+DTT Expected**	**$SNAP_f$+Dye Observed**	**$SNAP_f$+Dye+DTT Observed**	**% of Labeling**
SNAP-Surface 488	20539.1	20674.1	20538.5	20673.9	95.0
CBG-488-DABCYL	20539.1	20674.1	20538.6	20674.0	95.7
CBG-488-TQ2	20539.1	20674.1	20538.5	20673.9	95.6
SNAP-Surface 549	20768.2	20903.2	20767.8	20903.2	95.0
CBG-549-TQ3	20768.2	20903.2	20767.9	20903.6	95.0
CBG-549-QSY7	20768.2	20903.2	20768.0	20903.0	89.5
CBG-549-QC1	20768.2	20903.2	20768.2	20903.4	95.0
CBG-TF3	20408.2	20543.2	20407.3	20542.2	95.0
CBG-TF3-DABCYL	20408.2	20543.2	20407.6	20542.9	n.d.
CBG-TF3-TQ3	20408.2	20543.2	20407.4	20543.1	87.0
SNAP-Surface Alexa Fluor 647	20808.2	20943.2	20807.4	20942.9	95.0
CBG-AF 647-QC1	20808.2	20943.2	20808.0	20942.9	95.0
SNAP-Surface 647	20592.2	20727.2	20591.5	20726.8	95.0
CBG-647-QC1	20592.2	20727.2	20591.1	20726.7	95.0
CBG-TF5	20828.2	20963.2	20828.0	20963.5	95.0
CBG-TF5-QXL670	20828.2	20963.2	20828.1	20963.9	93.0
CBG-TF5-QSY21	20828.2	20963.2	20827.7	20962.6	64.5

[a] $SNAP_f$ protein was purified from $SNAP_f$-intein-CBD fusion constructs. The protein is released from the intein-CBD fusion partner in the presence of DTT.

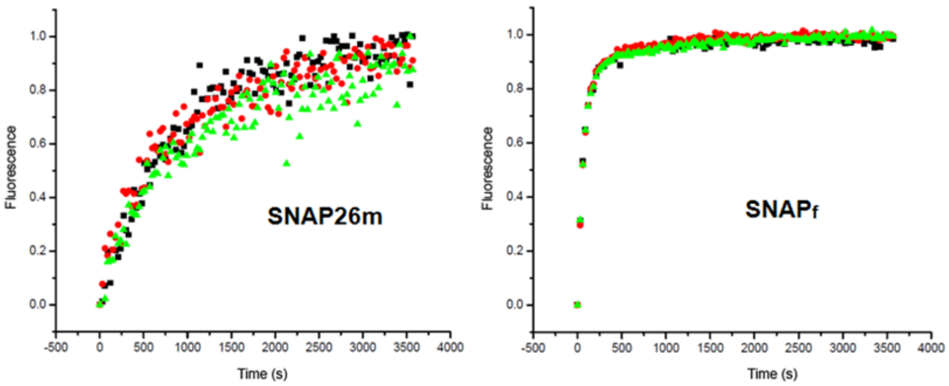

Figure S3. Kinetic measurements of $SNAP_f$ and SNAP26m towards the fluorogenic substrate CBG-488-TQ2. SNAP26m and $SNAP_f$ proteins were incubated at 3 µM final concentration at 37°C for 60 min in the presence of 30 µM CBG-488-TQ2. The change in fluorescence intensity was monitored using FLEXstation scanning fluorometer. $SNAP_f$ exhibited a pseudo first order rate of roughly 10-fold faster than SNAP26m. The individual rate constants for $SNAP_f$ and SNAP26m were determined as 350±3 and 38±5 $M^{-1}s^{-1}$, respectively.

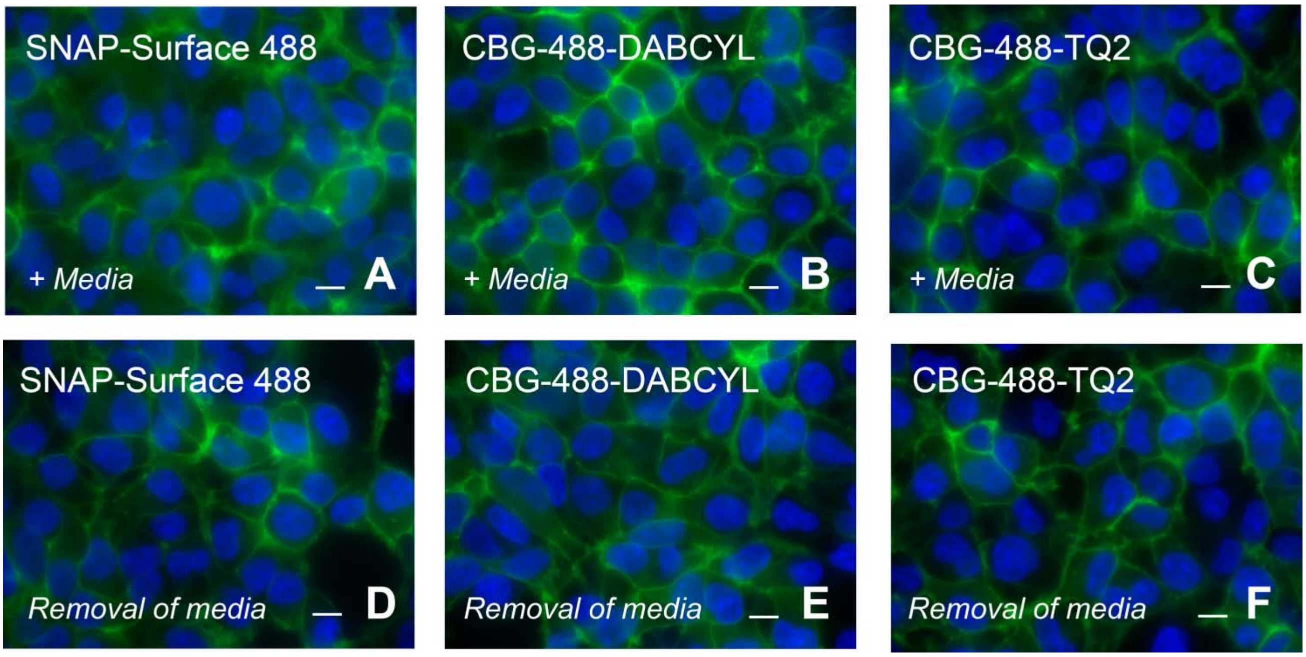

Figure S4. Comparison of SNAP-Surface 488, CBG-488-DABCYL, and CBG-488-TQ2 for labeling $SNAP_f$-EGFR in living cells. Live HEK 293 cells stably expressing $SNAP_f$-EGFR were incubated for 30 min at 37ºC with A) 1 µM SNAP-Surface 488, B) 1 µM CBG-488-DABCYL, or C) 1 µM CBG-488-TQ2. SNAP-Surface Block was added to the cells to a final concentration of 20 µM to inhibit further labeling. Images D), E), and F) were obtained after replacing the labeling media with complete growth media containing 20 µM SNAP-Surface Block. Images were taken on a wide field Axiovert 200M Zeiss microscope using a 63X objective and fixed exposure setting (400 ms). Cells were counterstained with Hoechst 33342 for nucleus (blue). Scale bars: 10 µm. Images were processed using AxioVision 4.7 software.

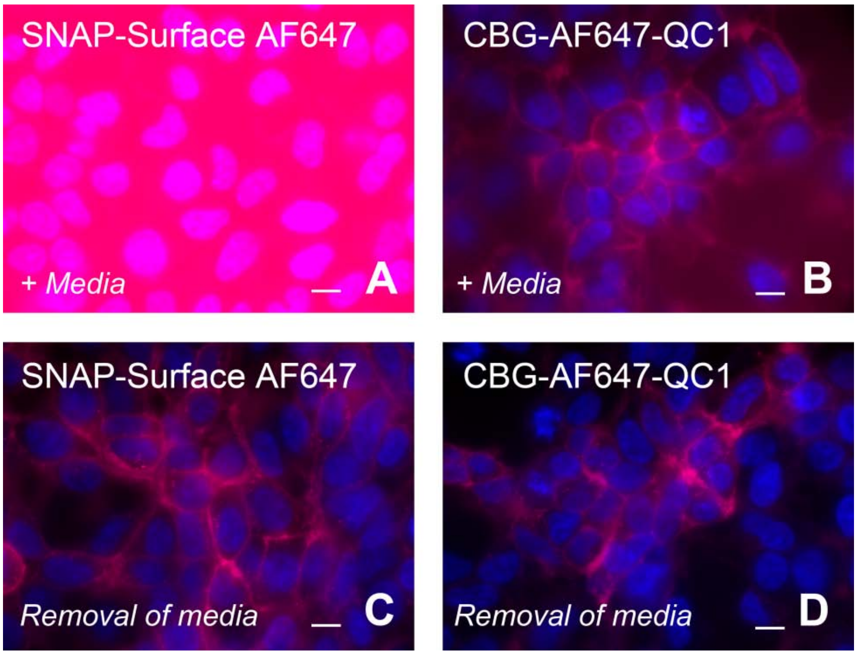

Figure S5. Comparison of SNAP-Surface Alexa Fluor 647 and CBG-AF647-QC1 for labeling $SNAP_f$-EGFR in living cells. Live HEK 293 cells stably expressing $SNAP_f$-EGFR were incubated for 30 min at 37ºC with A) 1 µM SNAP-Surface Alexa Fluor 647 or B) 1 µM CBG-AF647-QC1. SNAP-Surface Block was added to the cells to a final concentration of 20 µM to inhibit further labeling. Images C) and D) were obtained after replacing the labeling media with complete growth media containing 20 µM SNAP-Surface Block. Images were taken on a wide field Axiovert 200M Zeiss microscope using a 63X objective and fixed exposure setting (200 ms). Cells were counterstained with Hoechst 33342 for nucleus (blue). Scale bars: 10 µm. Images were processed using AxioVision 4.7 software.

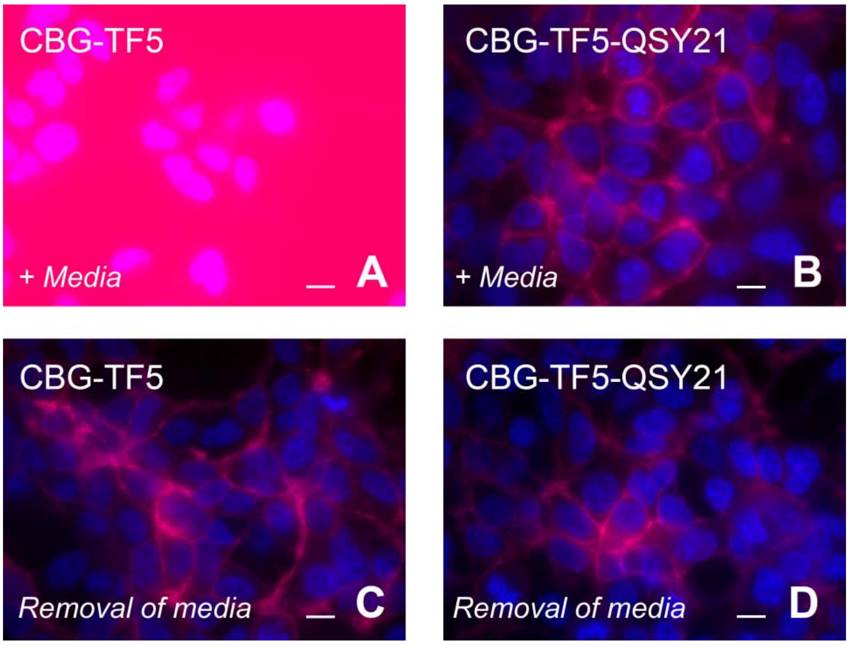

Figure S6. Comparison of CBG-TF5 and CBG-TF5-QSY21 for labeling $SNAP_f$-EGFR in living cells. Live HEK 293 cells stably expressing $SNAP_f$-EGFR were incubated for 30 min at 37ºC with A) 1 µM CBG-TF5 or B) 1 µM CBG-TF5-QSY21. SNAP-Surface Block was added to the cells to a final concentration of 20 µM to inhibit further labeling. Images C) and D) were obtained after replacing the labeling media with complete growth media containing 20 µM SNAP-Surface Block. Images were taken on a wide field Axiovert 200M Zeiss microscope using a 63X objective and fixed exposure setting (400 ms). Cells were counterstained with Hoechst 33342 for nucleus (blue). Scale bars: 10 µm. Images were processed using AxioVision 4.7 software.

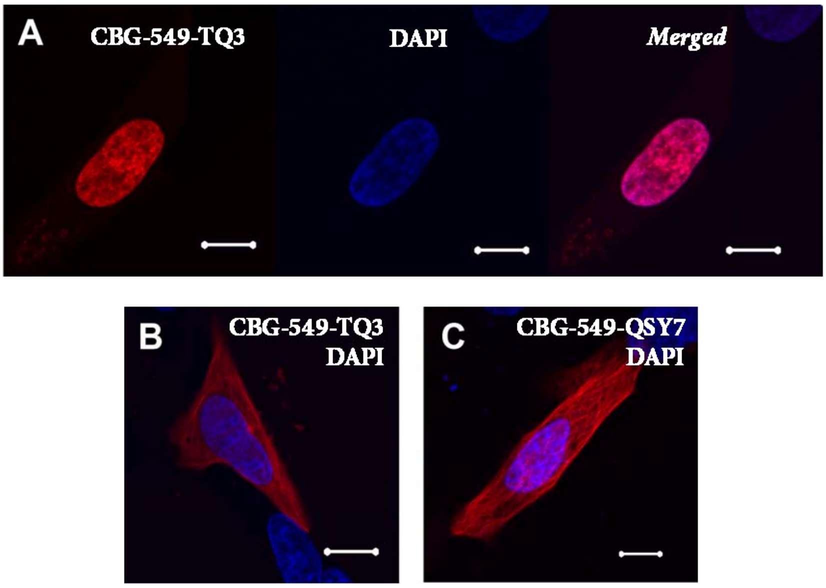

Figure S7. Live cell wash-free imaging of intracellular $SNAP_f$ fusion proteins labeled with fluorogenic substrates. U2OS cells stably expressing A) histone H2B or B) β-Tubulin tagged with $SNAP_f$ were incubated at 37ºC for 30 min with 100 µL of 10 µM CBG-549-TQ3. Glass beads (300 µm diameter, Sigma-Aldrich) were sprinkled onto the plate and rolled over the cells at the beginning of the incubation to allow permeabilization of CBG-549-TQ3 into the cells. Nuclear staining was performed with Hoeschst 33342 (blue). Images were taken on a Zeiss 510 Meta confocal microscope using 63X objective. C) U2OS cells transiently transfected with p$SNAP_f$-β-tubulin were cultured in 200 µL DMEM and labeled at 25ºC for 30 min with a labeling mixture containing 30 µL of DMEM, 1 µL Fugene 6 (Roche), and 1µL of 1 mM CBG 549-QSY7 (final concentration 4.3 µM) followed by confocal microscopy. Nuclear staining was performed with Hoeschst 33342 (blue). Scale bars: 10 µm.

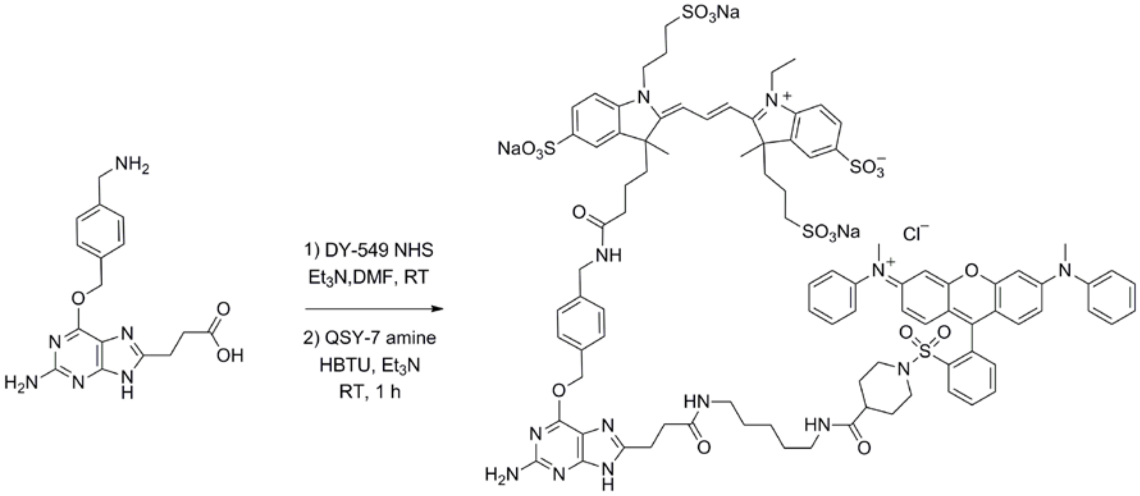

Scheme S1. Synthesis of CBG-549-QSY7. A solution of DY-549 NHS (5.0 mg, 5.1 µmol) and DMF (0.5 mL) was added to CBG-NH2 (1.7 mg, 5.1 µmol) and triethylamine (1.4 µL, 10.2 µmol) in DMF (0.5 mL). The reaction was stirred at 30°C overnight. QSY-7 amine hydrochloride (4.6 mg, 5.6 µmol), triethylamine (1.4 µL, 10.2 µmol), and HBTU (2.9 mg, 7.6 µmol) were added, and the mixture was stirred at 30°C for 2 h. The solvent was evaporated under vacuum. The purple residue was diluted in 1 mL water/acetonitrile (9:1) and the product purified by reverse phase HPLC. HPLC was performed on VYDAC 218TP series C18 polymeric reversed-phase column (22 x 250 mm, 10 µm particle size) at a flow rate of 20 mL/min using a water/acetonitrile gradient with trifluoroacetic acid (0.1%). Gradient: 5 min-5% ACN;10 min-10%; 15 min-50%; 20 min-75%; 25 min-95%; 27 min-95%; 30 min 10%. HPLC fractions were lyophilized. Yield: 5.3mg (2.7 µmol; 54%). ^{1}H NMR (500 MHz, DMSO-d_6) δ = 8.30 (d, *J*=13.1, 1H), 8.27 – 8.21 (m, 1H), 8.10 (dd, *J*=7.6, 1.3, 1H), 7.93 – 7.85 (m, 3H), 7.76 (d, *J*=5.1, 1H), 7.72 (d, *J*=11.9, 2H), 7.66 (t, *J*=8.8, 2H), 7.58 – 7.52 (m, 6H), 7.49 (d, *J*=8.4, 1H), 7.45 (d, *J*=7.5, 2H), 7.42 (d, *J*=7.4, 6H), 7.38 (d, *J*=8.1, 3H), 7.15 (d, *J*=8.0, 2H), 7.04 (d, *J*=9.4, 2H), 6.93 (s, 2H), 6.90 (d, *J*=9.1, 2H), 6.67 (d, *J*=13.2, 1H), 6.57 (d, *J*=13.3, 1H), 6.54 (s, 1H), 6.19 (s, 2H), 5.37 (s, 2H), 4.31 – 4.05 (m, 6H), 3.57 (s, 6H), 3.17 (d, *J*=11.7, 2H), 2.98 – 2.80 (m, 12H), 2.65 – 2.63 (m, 1H), 2.60 (t, *J*=5.3, 2H), 2.43 (t, *J*=11.8, 2H), 2.34 – 1.91 (m, 13H), 1.65 (s, 3H), 1.59 (s, 3H), 1.54 (d, J=11.7, 2H), 1.32 – 1.13 (m, 8H). ESI-TOFMS *m/z* 933.8112 $[M+2H]^{2+}$ (calcd for $C_{93}H_{103}N_{13}O_{19}S_5$, *m/z* 933.8127); UV (MeOH) λ_{max} 559 nm.
